# PET in the characterization of immune diseases and development of therapeutics

**DOI:** 10.1093/oxfimm/iqaf005

**Published:** 2025-05-24

**Authors:** Natasha Patel, Mats Bergstrom, Philip S Murphy, Juliana Maynard

**Affiliations:** School of Biomedical Engineering and Imaging Sciences, King’s College London, St Thomas’ Hospital, London, SE1 7EH, United Kingdom; OMID Molecular Imaging Consultancy, Uppsala, 75221, Sweden; Translational Science and Medicine, Immunology, Innovative Medicine, Johnson & Johnson, 50-100 Holmers Farm Way, High Wycombe, HP12 4DP, United Kingdom; Medicines Discovery Catapult, Mereside, Alderley Park, Cheshire, SK10 4TG, United Kingdom

**Keywords:** Positron Emission Tomography (PET), Molecular Imaging, Immune System, Immunology, Drug Development, Total Body PET, Biomarkers, Immune Cell Imaging, Inflammation, Immunotherapy, Radiotracers, Target Engagement

## Abstract

The immune system is a complex network of cells, tissues and organs that protects the body against harmful pathogens. Characterization of the immune system is essential for understanding the complex interactions underlying pathophysiology and providing insights to enable therapeutic targeting for modern drug development. Tissue and peripheral sampling report on important biomarkers, but may not adequately sample complex, heterogeneous systemic diseases. Imaging has been extensively used in the study of immune diseases, largely relying upon structural measurements of disease manifestation (e.g. X-ray for joint space narrowing in rheumatoid arthritis). These measurements are downstream from drug action, offering no insight into the intricacies of the immune system. Molecular imaging, particularly through Positron Emission Tomography has the potential to map the immune system at the whole-body level, providing non-invasive, quantitative readouts. Adoption of PET clinically and for drug development purposes for studying immune processes has been limited to date, lagging use in neuroscience and oncology. Emerging technical developments are likely to create new opportunities for immune system monitoring: (i) A broad set of clinical probes to study immune cells and associated processes are in development, (ii) The advent of TotalBody PET able to capture high-sensitivity measurements from all tissues with reduced radiation dose burden. This review explores the potential applications of PET for immune drug development, the technology advancements and suggests how adoption barriers can be overcome. The immune toolset of the future will likely demand an integrated approach, using tissue and peripheral readouts combined with immune-specific imaging.

## Introduction

### What is the purpose of studying the immune system?

Studies of the immune system aim to give us a better understanding of disease [1]. It gives us insight into the pathogenesis of disease, understanding how each disease develops, and progresses at molecular and cellular levels [2–4]. It is essential to understand how the immune system responds to different pathogens and the mechanisms involved in the response to how the immune system is activated and controlled [5]. In addition, understanding autoimmunity and investigating conditions where the immune system attacks the body’s tissues or reacts to harmless substances is also very important [6].

The use of different biomarkers and identifying a set of suitable biomarkers are crucial in identifying signatures and specific molecules that indicate the presence of disease [7]. They will significantly aid in the early diagnosis of immune disease, enabling the ability to treat at the early stages of disease onset [8]. Developing or available tissue-based tests that detect antibodies, antigens, and all immune components will be required to diagnose immune disease accurately [9], as well as using immune markers to track and monitor disease progression and, importantly, the effectiveness of treatments [10].

Whilst having a thorough understanding of the immune system in health and disease, it is also essential to have the correct targets to treat the disease effectively, and the discovery of new and novel immune components that new drugs can target is also important in the development of new therapeutics [11]. Recent years have seen the creation and development of vaccines that prime the immune system to fight off specific pathogens. Learning from alternative therapeutic strategies proven to be effective in other disease indications such as immunotherapies for autoimmune disease is important [12, 13].

### What are the challenges facing immunology drug development?

Precision and personalized medicine are essential concepts in the development of modern medicine. They help identify the right patient to respond to the aligned therapeutic regimen [14]. Using biomarker-driven therapy, developing therapies based on the presence of specific biomarkers, and combining all genetic, environmental, and lifestyle factors into personalized treatment planning will be necessary [15].

In immune disease there are a breadth of therapeutic platforms and a series of options to assess and develop the appropriate treatment option. Treatment options include monoclonal antibodies, designed to explicitly targeting disease [16] and cell-based therapies, including CAR-T cells that can modify and alter the body's immune response [17]. In addition, there is a need to further pursue the development of small molecules and biologics which can modulate the immune system [18].

Understanding the variability and the diversity of patient populations and their temporal evolution plus the individual immune response will be an essential component of how we know and can treat disease [19]; this includes ensuring that there is diversity in clinical trial populations to see how the treatments are working in different demographic societies and also in ensuring that in the development of therapies that these will also be accessible and effective in various parts of the world [20, 21].

### What tissue and peripheral markers of disease are available?

A series of tissue markers of disease are routinely available, allowing a greater understanding of immune disease. This includes histological analysis and immunohistochemistry, using antibodies to detect antigens and identify disease markers [22]. In addition, the ability to analyse the expression of a wide variety of genes enables the ability to perform and identify disease-related changes [23].

In addition to tissue markers, peripheral markers such as blood biomarkers that look at specific proteins and cytokines are key in the identification of signalling factors mediating and regulating immunity with the body’s physiological profile [24]. Metabolomics and profiling metabolites [25], sequencing DNA and RNA mutations [26], proteomics [27], and single-cell analysis are also important tools in the quest to really understand the broader picture and complex interplay of interaction in immune disease [28].

### Why do we need complementary, non-invasive, whole-body methods to understand immune disease?

Imaging techniques are crucial for non-invasive detection and follow-up of immune-related diseases [29]. Using imaging technology to visualize internal structures in the whole body and detect abnormalities is essential in understanding immune-related disease [30]. This includes imaging modalities such as Magnetic Resonance Imaging (MRI) [31] and Computed Tomography (CT) [32]. In addition, techniques such as Ultrasound employing sound waves to create images or Positron Emission Tomography (PET) that utilizes radioactive tracers to observe functional processes within the body are also very important [33].

The advent and introduction of whole-body PET as a technology has seen significant technological advancement in the ability to use PET as a non-invasive technology to understand the pathophysiology of immune-related diseases [34].

#### Current use of imaging to study the immune system in disease

**Figure 1.**
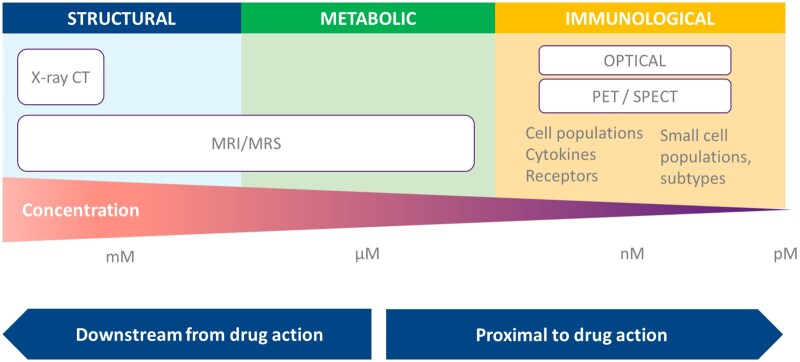
Indications for different types of imaging, the information that can be retrieved from each imaging modality, and the concentration typically required of any drug substance to detect on corresponding imaging technique.

Imaging technologies play a crucial role in the study of inflammatory, infectious and autoimmune diseases for diagnosis, disease management, treatment monitoring for clinical management and within clinical trials. Most imaging methods established for clinical use focus on the morphological and functional manifestations of disease. Due to the varied nature of diseases, a range of different methods have been established (Fig. 1). For example, in skin diseases where superficial inflammation can be visualized, evaluation can be done clinically and recorded using photography [35]. Analogously, endoscopy is routinely performed clinically and in clinical trials for the evaluation of the bowel wall in inflammatory bowel disease [36]. For inflammatory joint diseases multiple methods are routinely applied: structural evaluation by plain film X-ray to measure bone erosions and changes in joint space [37], MRI to measure disease associated synovitis [38], ultrasound to measure soft tissue structures and blood flow to identify active inflammation [39].

These established methods are widely available, generally at low cost within clinics or through radiology departments and have demonstrated clinical value. In clinical trials specifically, these methods also have established roles but have different utility across trial phases. For example, functional assessments may be more relevant to early phase trials where short term therapeutic response is interrogated. For later phase trials, morphological assessments with linkage to disease outcomes are used (e.g. X-ray).

Morphological assessment of immune diseases with imaging (MRI, CT, X-ray, US) will continue to be used broadly for clinical management and to deliver clinical trial endpoints:

- Low cost and accessible.
- Scalable to tens to hundreds of trial centres.
- Simple analysis/interpretation.
- Clinical value established.
- No or minimal radiation dose.

However, these methods are limited in their ability to define key aspects of the immune system and response to therapies, and critically only measure the downstream consequences of the aberrant immune system.

- Measure specific tissues/organs and have limited ability to characterize the heterogeneity of disease.
- Quantification is limited.
- Limited reversibility of morphology, particularly in terms of monitoring therapeutic interventions in short time frames.

Many of these methods have been established for decades. As we demand greater insights into the biology and progress with more complex therapeutic approaches, a new generation of measurement techniques will be required.

In particular, the pursuit of precision medicine, aiming to tailor interventions to individual patients, will necessitate a precise characterization of the immune system in disease at a patient level.

Tissue and blood-based measures continue to be the primary routes for molecular and immunological evaluation. Is there a role for advanced imaging techniques able to better support clinical evaluation of the immune system and guide our development of the next generation of immune targeting therapies?

### Molecular imaging has seen limited adoption clinically and for clinical trials

Molecular imaging conceptually holds promise to study the immune system with unique attributes compared to morphological and functional imaging modalities:

- Whole body evaluation captures the extent of systemic disease and can define disease heterogeneity.
- Quantitative measurements from specified tissues.
- Probes that are specific to metabolic processes or cell types can inform on different aspects of the immune system.

However, molecular imaging is not typically considered to be clinically useful broadly in the context of immune diseases. Given many clinical trial methods often parallel clinical use, it is unsurprising that adoption within drug development has also been lacking. This is despite evaluation of molecular imaging methods that have been established in areas like cancer imaging for decades. For example, FDG-PET as a pan-inflammation marker has been studied for over 20 years in a range of indications, including inflammatory bowel disease [40], rheumatoid arthritis [41], asthma [42], Sjogren’s disease [43]. Despite this extensive evaluation across a range of diseases, a broad role in clinical management or clinical trials has yet to be established.

Limited adoption of PET for clinical routine use and clinical trials is due to multiple reasons:

- Clinical value has not yet been established with PET tracers that are broadly available.
- The most widely available tracer FDG lacks specificity to immune processes.
- High procedure cost and limited availability at many centres.
- Radiation dose is limiting, especially for longitudinal evaluations.
- Complexity of analysis and lack of standardised analysis.
- Poor spatial resolution limits evaluation of small tissue structures, for example, skin, GI tract, small joints.

Clinical adoption of new imaging methods will require clear clinical value and practicality (including cost, availability) to be demonstrated. A likely starting point for new methods will be within drug development, where more innovative methods can be incorporated in small, specialized studies in early drug development. What technology advancements are required to shift from the current under-utilization?

- Whole body quantification to enable disease heterogeneity to be captured.
- Defined approaches to standardize derived measurements as trial endpoints.
- A range of molecule probes available to study different elements of the immune system.
- High quality imaging to enable the study of small tissue structures.
- Minimize radiation dose burden, particularly to scale use beyond small studies.
- Integration of imaging with tissue and blood-biomarkers make these complement, not compete.
- Linking immune imaging to structure and function.

The demands of drug development are further explored, with a focus on how imaging to date has provided unique insights.

#### Potential applications of imaging in immunology drug development

**Figure 2.**
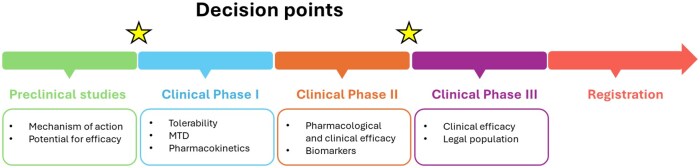
Phases of drug development and key decision points where PET imaging could be utilized.

Traditional drug development goes through a pre-defined path with preclinical explorations of mechanisms and validations of therapeutic opportunities. After evaluation of toxicity, the clinical phases are initiated with a structured path which traditionally is described as Phases I to III, each with its main objective (Fig. 2). The key parameters guiding the development are efficacy and side effects/toxicity.

This strict path entails some specific inherent problems:

- A weak link between pre-clinical and clinical studies.
- The dose is not well optimized, and many patients are under- or over-dosed.
- Attempts to obtain tissue samples are affected by low compliance.
- It is difficult to get good data on mechanisms and pathway effects.
- It requires efforts to convince physicians and patients to participate.
- Patients are often heavily pre-treated and refractory.
- Companions treatment are often not selected based on a complete understanding of mechanistic synergy.
- There is an overall high failure rate and many late and expensive program failures.

In order to modernize drug development, the ‘Critical Path Initiative’ from FDA suggested a less strict path and puts an emphasis on mechanistic understanding including more reliance on biomarkers [44]. It is in line with this that we here focus on Molecular Imaging as one type of biomarker with its specific advantages but also its hurdles and shortcomings.

**Figure 3.**
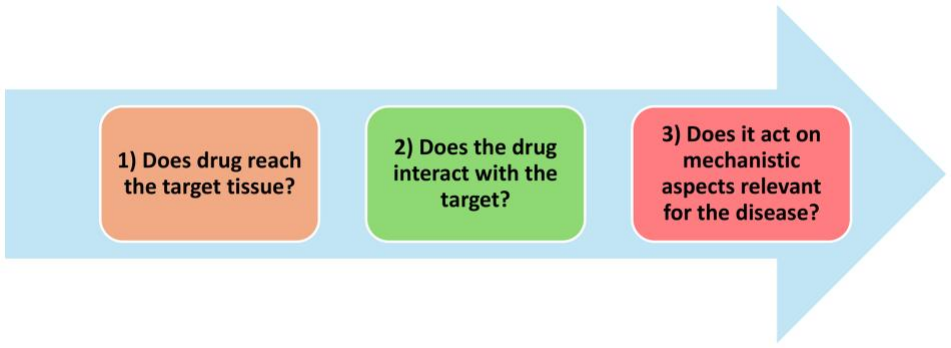
The three pillars of drug development.

An evolving insight regarding failures of drugs to reach the final stage of registration and introduction into the market has been framed as the ‘Three Pillars in Drug Development’ (Fig. 3) [45]. It was realized that many failures could be attributed to inferior knowledge regarding:

- The drug’s access to the site where the target resides.
- The drug’s interaction with the target.
- The drug’s induction of a therapeutically relevant pharmacological action on cells/tissues/organs.

We believe that Molecular Imaging under certain conditions may contribute to the understanding of these questions and discuss here some of the instances whereby other methods have shortcomings, highlighting where Molecular Imaging can give valuable information.

### Drug biodistribution

Therapeutic agents may be administered to the subject via one of a range of routes: direct injection into tissue, intravenous injection, deposition into the skin, inhaled, orally ingested, etc. In a large fraction of cases the aim is for the drug to reach the general circulation and from there access the site of action. This means that it is in most instances desired to explore plasma PK and this is readily achieved through blood sampling.

However, this is not the same as proving access to the target space which may reside in the interstitium or intracellularly. In many cases the PK-profile at the target site can be inferred from plasma PK, but there are cases where this extrapolation is difficult, for example, for targets protected by the Blood-Brain-Barrier (BBB) or the drug is affected by cellular efflux systems.

In some of these cases there may be a role for a biodistribution study using PET with a radiolabelled drug.

The BBB with its tight junctions in the capillary bed and a set of efflux systems may effectively prevent drug’s access to the brain tissue. This is valid for all sizes of molecules from small organic compounds to large protein drugs.

Most small molecules are organic compounds containing carbon, and in many cases there are routes for radiolabelling with ^11^C or ^18^F (PET radionuclides with 20 min and 2 h half-life, respectively). This labelling ensures unchanged distribution and binding properties and following its distribution gives a means to study drug biodistribution. This has been applied in a number of studies of neuro-active pharmaceuticals [46–51]. There are usually no alternative techniques since direct access to the brain is restricted and sampling of CSF (cerebrospinal fluid) is invasive and has questionable relevance to brain interstitium and cellular space.

For such brain PET studies, present PET scanners are usually adequate, with most of them having an anatomical coverage which is sufficient. However, in some cases there may be an advantage to also explore the general body distribution. This may relate to a desire to understand if the drug is accumulating in organs of sensitivity, indicative of risk for side effects. Present conventional PET scanners need to cover the body by sequential acquisitions over 5–7 body sectors, which takes in the order of 30–40 min, whereupon a new set of scans can be performed. This means first of all that there is a very sparse temporal sampling of tissue PK, and secondly that the sensitivity is severely reduced compared to the option given by WBPET (Whole Body PET).

This illustrates also the advantages of WBPET in instances where whole body biodistribution of drugs are directly desired. Examples of this are protein pharmaceuticals like peptides or antibodies and antibody fragments where degree and rate of extravasation in different organs and pathological tissue is of interest [52–54]. A PET study with temporal acquisitions may support an understanding and also give data for a better definition of a PBPK-model as demonstrated by the Albudab study by Thorneloe KS *et al*. and Sepp A *et al.* [55, 56].

### Microdosing

A regulatory concept called ‘microdosing’ has been approved by US and EU regulatory agencies, in line with the critical path initiative [57–59]. This means that human exploration of new drugs can be performed at microdoses, in general up to 100 ug for small molecules and 30 nmol for protein drugs, with a reduced preclinical package including toxicity evaluation. The latter typically means a 2 days and 2 weeks clinical observation and histology in a single species. Genotox evaluation is not required but the issue of immunogenicity for proteins is still uncertain. This concept aims for early biodistribution to be evaluated in a human setting sufficiently early to allow a decision on progress or termination due to unfavourable PK properties.

This concept is also especially important for the further development of specific PET probes which typically are administered at microdosing levels, but also gives the option to study PET-labelled drugs for entities under development with the additional information of whole-body biodistribution as a complement to plasma PK. Under certain conditions such a study may also suggest targeting to specific tissues indicative of target engagement.

As mentioned above, the use of WBPET in such settings is advantageous with simultaneous dynamic whole-body imaging and the increased sensitivity may be important to keep the mass dose low if required due to the specific activity.

### Inhaled drugs

The inhalation route of drug administration is important but is also associated with some challenges. Depending on physio-chemical properties of the drug in combination with the inhalation device, the deposition pattern can vary considerably, from a mostly oral/nasal deposition to full coverage of the lung. The deposition pattern is also likely to be affected by the patient characteristics, for example impaired lung function globally or regionally. Thus, in inflammatory lung disease the drug access to the critical structures is fundamental, and there are usually limited alternatives to record drug deposition in lung. Lavage studies are performed but they have limited anatomical discrimination and are invasive in their nature. Lung biopsies are usually avoided due to increased risks.

Here Molecular imaging may fill an important gap, allowing excellent anatomical delineation of deposition and disposition, the latter by monitoring the lung radioactivity pattern dynamically [60–63]. As in many other applications, a PET scanner with extended anatomical coverage is an advantage.

### The specifics of ADCs and radio-theranostic delivery

**Figure 4.**
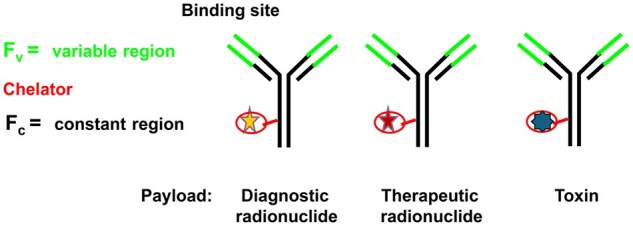
Generic methods of attaching radioactive or therapeutic payload to antibodies.

Antibody-drug-conjugates (ADC) and antibody-radioactivity conjugates (ARC) are well established in oncology as means to target the treatment agent to specific tissues or cells by a targeting antibody or other scaffold coupled with the therapeutic agent (Fig. 4).

Although these concepts are well suited also for infectious and inflammatory diseases, there are fewer examples where Molecular Imaging has been used for such therapeutics in this field.

Molecular imaging can here have an important role to prove targeting and to explore the amount of delivery of the payload by interpretation of the antibody scaffold’s homing and possible internalization by the targeting mechanism [64, 65]. It is seldom possible to have the payload, attached to the carrier, radiolabelled. The payloads are often small molecules which could be labelled with the short-lived radionuclides ^11^C and ^18^F, but the whole process of generating the conjugate and also the long monitoring times needed typically prevent such an approach.

### Liposomal deliveries

**Figure 5.**
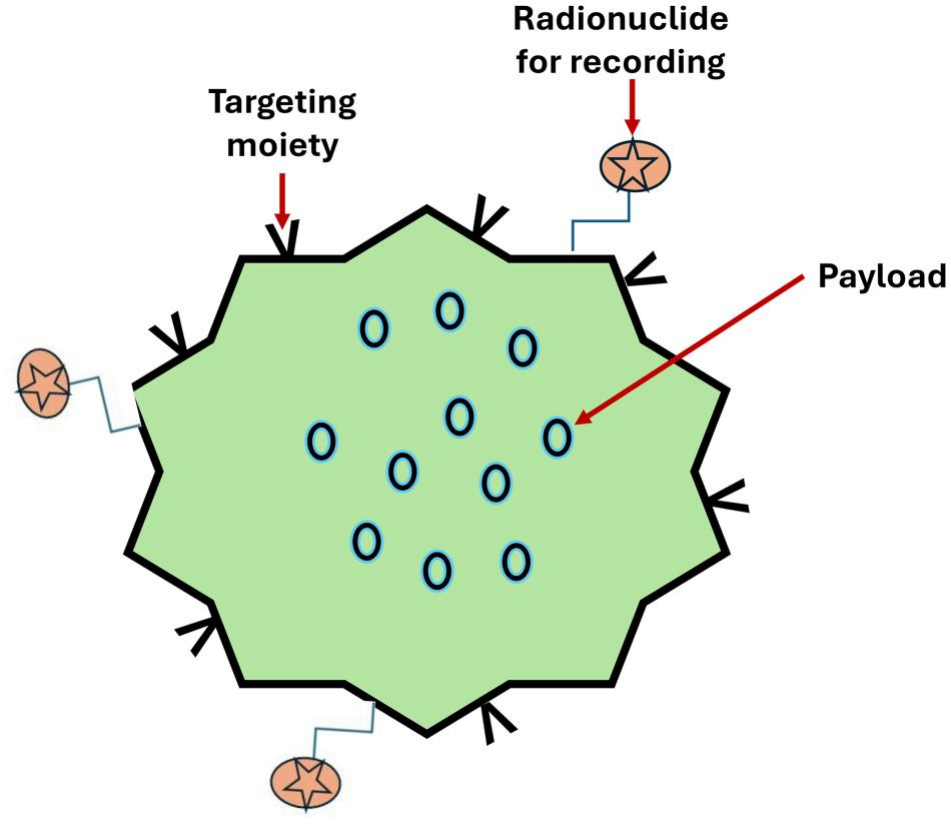
Introduction of payload and radioactive probes to liposomes.

To achieve some targeting and to improve the PK of therapeutics, they may be encapsuled in nanoparticles like liposomes (Fig. 5). Each nanoparticle may contain a large amount of payload, and the nanoparticle may have targeting properties, for example by having targeting moieties attached to the surface. When binding to the targeted cells, the payload may be released at the instance of cell attachment or may be internalized, guided by the targeting, to release the payload intracellularly.

The targeting ability may be monitored by having the nanoparticle radiolabelled for a PET biodistribution study.

This is exemplified in a preclinical study by Cheng S-H *et al.* [66] where the distribution of ^89^Zr-labelled liposomes with an antibacterial payload was monitored by PET in a rodent infection model and the payload recorded in excised tissues at the end of the study using MALDI.

Model-based analysis allowed the liposome delivery to be extracted and found to correlate well with deposition of payload. The results support the notion that in a human PET biodistribution study the delivery of payload to different tissues can be estimated. Such a study is best performed by the use of WBPET with its simultaneous whole-body coverage and increased sensitivity.

### Tracking of therapeutic cells

Different types of therapeutic cell strategies have been developed or are under development, most prominently CAR-T-cells by which syngeneic T-cells are extracted and externally modified for specific targeting and re-injected. Cases of excellent therapy have been described, mostly in oncology but also in autoimmune diseases [67].

In order to prove targeting, different strategies have been employed for radiolabelling of CAR-T-cells with the aim to monitor their distribution and homing to pathological tissue using external Molecular Imaging [68–70].

For the optimal quantitation, the best resolution and allowing longer observation times, PET radionuclides like ^89^Zr have been used. Radiolabelling have been probed with ^89^Zr-oxine and ^89^Zr-DFO, with slightly different degrees of cellular incorporation but also slightly different biodistribution.

There are significant limitations in the amount of radioactivity which can be incorporated into cells, due to progressive ^89^Zr efflux, a limitation in the number of cells that are labelled, and most of all there is a limitation in the number of cells which accumulate in a certain tissue. For this reason, we believe that such cell tracking evaluations in humans will require WBPET with its increased sensitivity and whole-body coverage.

### Target engagement

A critical aspect and the second ‘Pillar’ require the demonstration of target engagement. This is in general implying a demonstration of a sufficient degree of interaction with the target to suggest it being adequate for the desired pharmacological effect. For agonist drugs it may be sufficient to induce 5–10% occupancy on a target receptor, while for antagonist drugs it may require 90–95% occupancy of a receptor or inhibition of an enzyme to ensure clinically adequate inhibition of a pathway.

Demonstration of target engagement in tissues obtained at biopsy is not always trivial and except for problems of obtaining representative tissue there may be methodological issues with quantitation of target engagement.

In the field of neuroscience, PET has long been used for target engagement studies utilizing a target specific PET-probe [71–73]. The fraction of free receptors or enzymes are extracted by modelling of the PET-signal in relation to plasma radioactivity or the PET signal in a reference tissue devoid of targets. One or a few repeat studies are performed after drug challenge and the dose-dependence or temporal course of the target engagement are deduced. This concept has proven very useful in the neuroscience field, but less well utilized in other human diseases.

The option of performing target engagement studies with antibodies is nicely demonstrated in the HER3 study utilizing PET with ^89^Zr-labelled anti-HER3 antibody [74].

### Monitoring effects on cells and tissues

Therapeutic effects imparted by drugs on cells or tissues can be mechanistically proximal to the target interaction like effects on signalling pathways, intermediate-like effects on cellular function or distant-like effects on tissue physiology, for example blood flow. It is often desirable to observe effects proximal to targeted mechanisms, but these effects should also be relevant for a clinical endpoint. The most common example for Molecular Imaging for response monitoring is FDG evaluation in cancer treatment where the measured entity, the glucose utilization pathway, has been shown in many cases to translate into consequences on tumour growth rate or change in tumour size.

In inflammatory or infectious diseases FDG has been used for treatment monitoring during drug development but not to the same extent and with the same impact as in oncology.

Beyond FDG there are very sparse examples of Molecular Imaging for assessment of treatment response.

A line of interest is the approaches to characterize drug effects on specific cell components like macrophages, T-cells or neutrophils by cell specific probes. Studies in biopsy samples have indicated that the best biomarker of response in Rheumatoid Arthritis (RA) is the population of macrophages [75]. It is therefore of interest to follow the emerging validation of macrophage specific imaging in RA using probes like Folate-receptor-A-targeting [76], or the TSPO tracers [77].

Several approaches to visualize and monitor T-cells are promoted in oncology but could potentially have a role also in inflammatory diseases including COVID effects in the lungs [78–80]. Below in the text is further elaborated upon tracers for activated T-cells: ^18^F-CD8-scaffolds and ^18^F-AraG. The publication on ^18^F-AraG in long COVID [80] indicates a few specific advantages of molecular Imaging: the option to observe multiple organs and substructures in the same setting, and the potential to repeat the studies and have quantitative values for the assessment of changes with time or in relation to treatment or natural history of the disease. However, we must emphasize key aspects of quantitation as described below, for example to ensure proper concerns to potential changes in tracer blood clearance. Furthermore, it is important to consider that uptake in a specific tissue may be dependent upon perfusion and extravasation which may be changed by pathology.

Given these findings, molecular imaging could play a crucial role in tracking immune responses in post-viral syndromes. Future research should explore how PET-based T cell tracking could confirm persistent inflammation from viral reservoirs, refine biomarkers for disease progression, and assess its application in other chronic inflammatory conditions. Expanding PET-based immune imaging across these domains could significantly enhance our understanding of chronic inflammation and inform precision medicine strategies in immunology. For instance, the potential to visualize T cell activation in avenues such as neuroinflammation could lead to better understanding the role of T cells in neurodegenerative diseases like multiple sclerosis.

Specific imaging of neutrophil recruitment using a neutrophil elastase PET tracer has shown interesting results in COVID infected lungs in humans and ARDS models in pigs and rats [81].

### Observations of adverse effects

Many drugs can induce adverse effects that may limit their use or necessitate restrictions on doses and dosing schedules. In some cases, the organs at risk may be identified during drug development, prompting specific monitoring efforts. Molecular imaging can sometimes be employed for patient monitoring, particularly to detect these adverse effects. For example, inhibition of the PD1-PDL1 pathway has been linked to arthritis, and FAPI-PET imaging has successfully demonstrated this phenomenon [82]. Similarly, FAPI tracers can be used to monitor drug-induced lung fibrosis, highlighting the potential of molecular imaging in managing adverse effects.

### Technological advancements

The effectiveness and growing potential of molecular imaging in diagnosis, as well as monitoring therapeutic and adverse effects within immunological drug development is largely driven by ongoing technological advancements. Innovations in molecular probes, the development of Total Body PET (TBP), and improvements in analysis and modelling have all significantly enhanced our ability to understand immune responses in the context of disease.

Technological advancements including molecular imaging have significantly enhanced our understanding of the immune system in the context of disease [83–85]. This section delves into three key areas of innovation: the development of a range of molecular probes, the advent of Total Body PET, and advancements in analysis and modelling.

### Range of molecular probes

**Figure 6.**
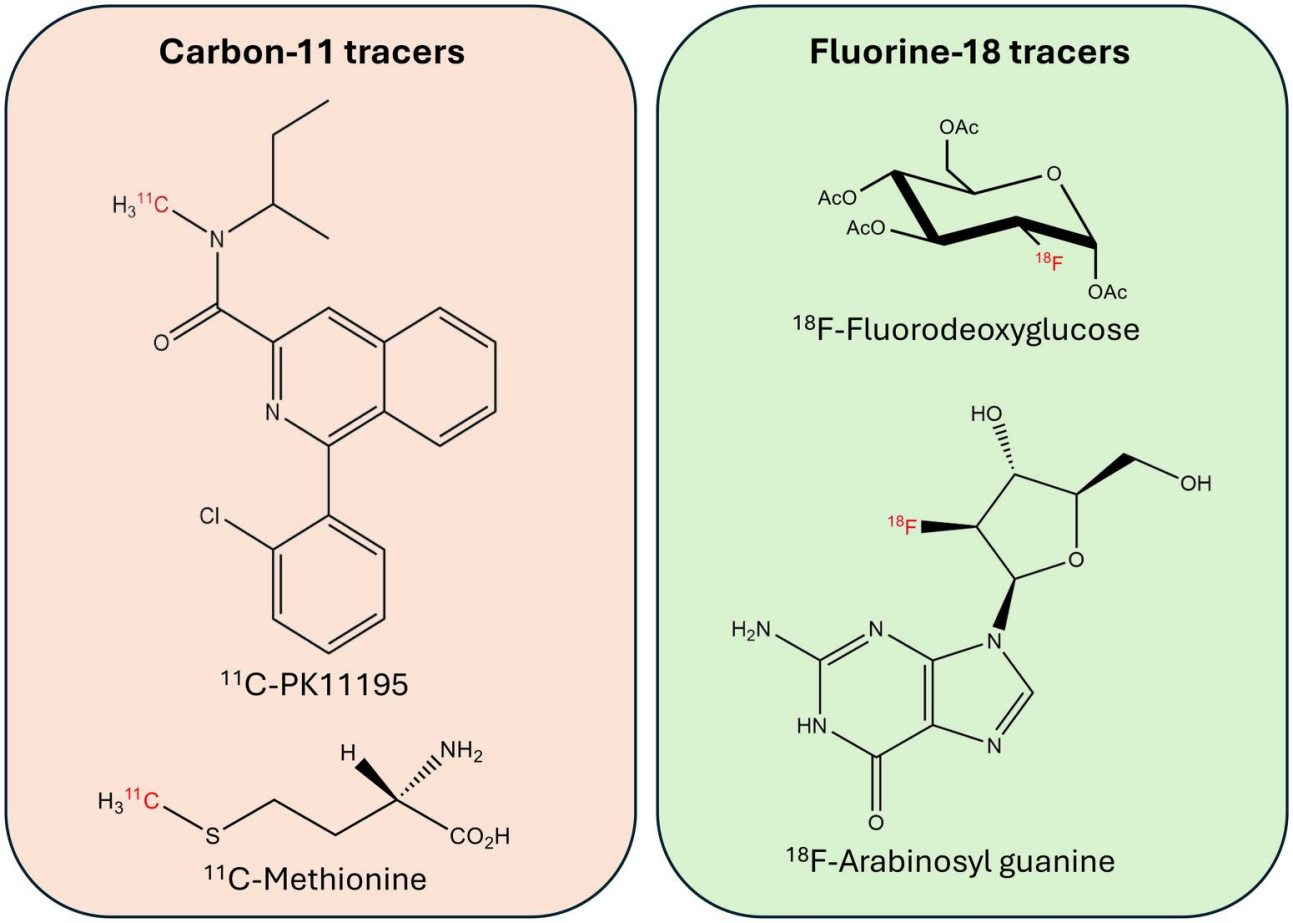
Examples of carbon-11 (^11^C) and fluorine-18 (^18^F) tracers frequently used in PET imaging. ^11^C-PK11195 targets the translocator protein (TSPO) and has been used to image activated microglia, which are involved in neuroinflammatory processes. It has applications in studying neuroinflammation in diseases like multiple sclerosis and Alzheimer's disease. ^11^C-methionine primarily targets amino acid metabolism. It is used in imaging inflammatory cells that are actively synthesizing proteins. It is useful in studying immune cell activity in tumours and inflammatory lesions. ^18^F-fluorodeoxyglucose (^18^F-FDG) is the most widely used PET tracer in immunology and oncology. It highlights areas of high glucose uptake, such as inflammatory sites where immune cells (like macrophages, neutrophils and lymphocytes) are active. ^18^F-arabinosyl guanine (^18^F-AraG) targets deoxycytidine kinase (dCK). This tracer is used to image T-cell activation. It has been used in research to monitor the immune response, particularly in the context of cancer immunotherapy.

A significant technical advancement in radiochemistry, particularly in the direct labelling of molecules with carbon-11 (C-11) and fluorine-18 (F-18), has revolutionized the development of molecular probes used in positron emission tomography (PET) by providing simple and quick production of probes (Fig. 6) [86–88]. These advancements have enabled the creation of highly specific and effective imaging agents, enhancing the accuracy of diagnostic techniques [89]. The development of radiochemistry has also led to improvements in specific radioactivity—the amount of radioactivity per unit mass of a radionuclide—which is crucial for obtaining high-quality images while minimizing patient radiation and compound exposure. This progress is illustrated in the accompanying Fig. 6, which highlights the facile synthesis of a molecular probe labelled with C-11 and F-18, demonstrating the efficiency of modern radiochemical techniques.

**Figure 7.**
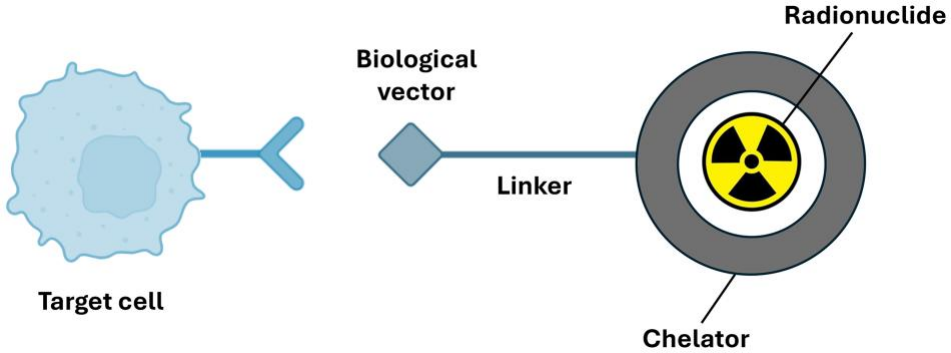
Components of a molecular probe for targeting receptors on cells of interest.

The construction of a molecular probe in a radiopharmaceutical involves selecting a biological target associated with a disease and choosing a specific targeting moiety, such as an antibody or peptide, that binds to this target (Fig. 7) [90]. A suitable radioisotope is then selected based on its half-life and emission properties, typically these are: gallium-68, zirconium-89, fluorine-18, technetium-99m, copper-64. The targeting moiety is chemically conjugated to the radioisotope using appropriate linker chemistry. The development of diverse molecular probes has revolutionized molecular imaging, providing unprecedented insights into immune system dynamics.

For instance, radiolabelled proteins, such as antibodies targeting immune cell markers such as CD4, CD8, and PD-L1 enable the detailed imaging of T-cell populations and their distribution within tissues. These probes can highlight areas of immune activation, suppression, or evasion, which are critical in diseases such as cancer, autoimmune disorders, and infections. Fluorescent probes and contrast agents are also widely used in optical imaging, allowing for real-time visualization of immune responses in live animals and, increasingly, in clinical settings.

Imaging the immune system with molecular probes involves targeting specific immune markers to visualize immune activity and responses. For instance, CD8-targeted probes can be used to image cytotoxic T cells, which play a crucial role in the body's defence against infections and cancer [91]. By using radiolabelled antibodies or peptides that bind specifically to CD8 molecules, PET or SPECT imaging can provide detailed images of CD8 + T cell distribution and activity. CD8 T cell distribution can be a potential biomarker for immune checkpoint inhibitor (ICI) response, predicting cancer immunotherapy response at an early stage, and therefore inform on effective treatment plans. There are currently a few investigated tracers for CD8, predominantly of the immunoPET type (antibody-based tracers). This includes an F-18 labelled nanobody probe produced by GE, ^18^F-GEH200521, currently undergoing phase I clinical trials as well as the anti-CD8 minibody, ^89^Zr-Df-IAB22M2C, which has undergone patient clinical trials [92]. An exemplary peptide-based probe is ^18^F-arabinosyl guanine (AraG) which has been used for pre-clinical and clinical imaging of rheumatoid arthritis, and oncology, amongst other diseases, for the targeting of CD8 + T-cells [93, 94]. Due to the relatively short half-life of F-18, this tracer could be used for same-day and longitudinal imaging, therefore benefitting patient treatment monitoring, and consequently tailoring treatment plans.

Fibroblast activation protein inhibitors (FAPI) are versatile imaging agents capable of detecting lesions across a wide range of diseases. They are particularly effective in oncology, where they highlight cancerous tumours, but they also show lesions in inflammatory and immune conditions such as rheumatoid arthritis [95, 96]. Beyond these, FAPI imaging has been reported to identify disease activity in cardiovascular disorders, fibrosis, and even infectious diseases, making it a powerful tool for diagnosing and monitoring various pathological conditions. FAPI probes, labelled with radioisotopes, enable the visualization of tumour microenvironments and inflammatory processes in various diseases through PET imaging, offering insights into immune system dynamics and disease pathology [97].

FAPI has recently emerged to be a superior contender for ^18^F-FDG in the clinic. For example, head-to-head comparisons of ^18^F-FDG and ^68^Ga-FAPI across a variety of different cancers with PET/CT, showed improved imaging sensitivity, especially amongst pancreatic and gastric cancer imaging [98, 99]. FAPI has demonstrated a crucial role in diagnostic applications, however, it is important to highlight the emerging radiotherapeutic value observed. In a pilot study, Lindner *et al.* have shown that patients with metastatic breast cancer who were administered ^90^Y-FAPI-04, a beta minus emitter, had exhibited rapid internalization of the radiotracer to FAP-positive metastatic tumours (10 min post-administration), and fast clearance from the body, whilst showing improved clinical symptoms: a reduction in pain symptoms, and no side-effects were observed, especially hematoxicity [100]. In a clinical study by Baum *et al.*, patients with diverse metastatic adenocarcinomas demonstrated significant tumour uptake and retention of the radiopharmaceutical in all patients treated with ^177^Lu-FAP-2286 [101]. These studies illustrate the potential FAP-targeting has in diagnosis and treatment. With respect to therapeutic studies, the combination of providing a therapeutic FAPI tracer with diagnostic imaging with ^68^Ga-FAPI tracers, serves to provide a vital way to select patients for this type of treatment, possibly for individual optimization of dose and to monitor disease progression, more so to a greater level of accuracy with ^68^Ga-FAPI TB PET/CT imaging.

The promising pre-clinical and clinical results of FAPI radionuclide imaging has led to the gradual interest of what FAPI could exploit when using TBP. To date, there has been one clinical example of a FAPI tracer using TBP in China, ^68^Ga-FAPI-04, investigating total-body kinetic modelling and dynamic in pancreatic and gastric cancer using a TBP uExplorer scanner [102]. This total body parametric imaging study showed improved lesion contrast which provided excellent tumour visualization, characterization and quantification, all aiding the downstream evaluation of the most effective treatment course. This work, as well as the PET/radiotherapeutic studies, highlights how FAPI in TBP can serve as a promising imaging method for early diagnosis and for monitoring disease progression.

### The advent of total body PET

**Figure 8.**
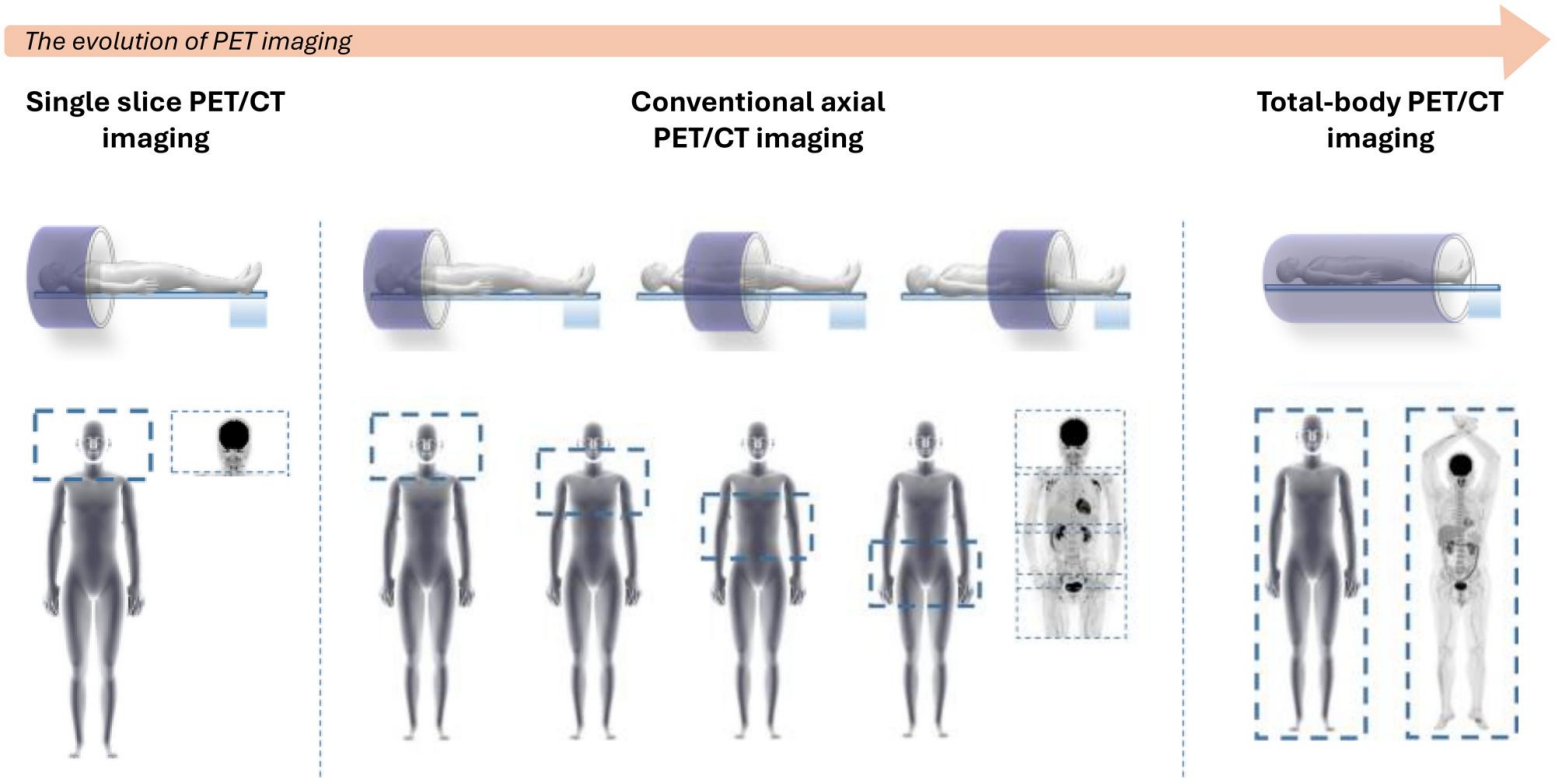
Evolution of PET imaging from single-slice imaging, to conventional axial PET/CT imaging, leading to the current state-of-the-art total body PET/CT imaging. Figure adapted from Sun *et al*. [103].

The first PET systems, introduced in the 1970s, had a single ring of detectors, enabling a single slice of the body to be recorded (Fig. 8). The use of radiotracers such as ^18^F-FDG (fluorodeoxyglucose) allowed for detailed imaging of glucose metabolism, revolutionizing the study of brain function and tumour metabolism.

The 1980–1990s saw PET imaging become more widespread in clinical settings, particularly in the fields of oncology, neurology, and cardiology. The next generation of scanners had two rings with two direct and one in-between slice. Advances in computer technology and image reconstruction algorithms improved image quality and diagnostic accuracy, making PET a valuable tool in medical diagnostics.

The integration of PET with CT in the 2000s led to the development of PET/CT scanners, which combined functional and anatomical imaging. This hybrid imaging technology provided more precise localization of metabolic activity, significantly enhancing the diagnostic power of PET and expanding its clinical utility.

Further expansion of the detector coverage in PET/CT scanners lead to a 10–15 cm axial coverage and now with what we define as Total Body PET (TB-PET), Whole Body PET (WB-PET) or Large Field PET (LF-PET) the axial coverage has been extended to 80–190 cm. The geometric resolution is of the order of 5–7 mm, meaning that an infinitely small structure is still represented as being 5–7 mm (Full Width at Half Maximum, FWHM) and two structures need to be separated by more than 5–7 mm to be seen as separate. Accurate quantitation requires structures typically with a smallest diameter of 10–15 mm. The sensitivity has been greatly increased by the larger angular coverage to be 4 to 16 times better than the previous scanner generation. This means that very low levels of radioactivity can now be imaged. This in turn allows studies to be performed with much lower amounts of administered radioactivity and hence a reduced radioactivity exposure to the subject. This is especially important in the study of healthy individuals or subjects with less severe diseases where the typical radiation dose exposure limit is 10 mSv. In severe diseases like oncology larger exposures are acceptable with, for example 40 mSv exposure. Another opportunity is the ability to follow the radioactivity in a subject for longer times where the gradual decay minimizes the amount of radioactivity in the body.

PET has long been a cornerstone of molecular imaging, but the recent development of TotalBody PET has expanded its capabilities dramatically. Traditional PET scanners are limited to imaging a single body region at a time, but TotalBody PET can image the entire body simultaneously. This breakthrough allows for comprehensive tracking of immune responses and disease processes throughout an entire organism, capturing dynamic interactions in real-time. TotalBody PET offers several advantages for immunological studies:

- High sensitivity and spatial resolution: enabling the detection of low-abundance molecular targets.
- Measurement of systemic immune responses: essential for understanding diseases that affect multiple organ systems.
- Ability to perform whole-body imaging with a single scan: reduces radiation dose to patients and improves throughput in clinical settings.

For dynamic or sequential biodistribution studies a simultaneous whole-body coverage is a great advantage but the increased sensitivity of WBPET may also be a significant advantage. The relation between amount of radioactivity given (MBq), which governs the quality of the images, and the mass of unlabelled compound in the injectate (nmole or ug) is given by the specific activity (MBq/nmole). For small organic molecules labelled with short-lived radionuclides like ^11^C or ^18^F, the specific radioactivity is such that an imaging-relevant radioactivity administration (100–1000 MBq) is associated with a mass of 10–100 µg. Such mass doses may in some instances be sufficient to induce pharmacological effects and there may be a desire to further reduce the mass, implying also a reduction in the administered radioactivity. Here the increased sensitivity of WBPET by a factor of 4–16 compared to conventional PET may be critical.

For protein pharmaceuticals with long plasma residence and slow extravasation, radionuclides like ^89^Zr with half-life of 3.3 days may be preferred. However, this radionuclide is associated with a proportionally high radiation dose to the subject. Typically, such a study with 15 MBq and a few nmoles of mass is associated with a radiation dose of 10 mSv which from radiation regulations is the exposure limit for healthy volunteers and subjects with more benign diseases [55]. With this low amount of radioactivity, the image quality is greatly impaired and gives reasons to utilize the improved sensitivity supplied by WBPET.

Most of the methods used to characterize functional effects on cells and tissues are using a simplified scanning scheme with a single time point of acquisition. This, hence, can be performed with the present smaller field scanners and sequential scanning across the boy. However, with anatomically extended diseases like RA, there is a significant gain in sensitivity by using WBPET. For each anatomical position, the extended angular coverage contributes with about a factor of 4. The option to cover simultaneously the whole body, rather than sequentially cover the body with about 4 sectors means an additional gain with a factor of about 4, hence an overall gain by about a factor of 16 which is substantial with respect to improving the image quality by a reduced noise level of about a factor of 4 (square root of 16). Alternatively, this increased sensitivity can be utilized to lower the amount of administered radioactivity and hence the radiation exposure, a factor which can be especially important if multiple scan sessions are desired, for example to follow the drug effect with time.

### Analysis and modelling

PET typically supplies quantitative values as Standardized Uptake Values (SUV), this is the ratio between the radioactivity concentration within a certain area compared to the entire body. This quantitative approach allows for precise monitoring and comparison of PET probe residence across different regions.

Advancements in data analysis and computational modelling have greatly enhanced the utility of molecular imaging. Sophisticated algorithms and machine learning techniques are now being applied to interpret complex imaging data, enhance image quality, providing deeper insights into pathophysiological behaviour and disease mechanisms [104].

Recent advancements in the analysis of radioactive metabolites, particularly through the integration of high-performance liquid chromatography (HPLC) and advanced modelling techniques, have significantly improved our understanding of the pharmacokinetics and biodistribution of molecular probes *in vivo*. The direct *in vivo* measures provided by PET give the total radioactivity, irrespective if it derives from intact compound or metabolites thereof. Separation can partly be made by further analyses. By precisely quantifying and characterizing the metabolites of radiolabelled compounds, researchers can better assess the stability and specificity of these probes, ensuring they target the intended biological pathways with minimal off-target effects. This detailed metabolic analysis, when coupled with kinetic modelling, allows for the optimization of molecular probes for PET imaging, leading to more accurate diagnostics and personalized treatment strategies. Consequently, these technological advancements not only enhance the quality of imaging but also provide critical insights into probe behaviour, ultimately benefiting patient care by enabling more targeted and effective interventions.

**Figure 9.**
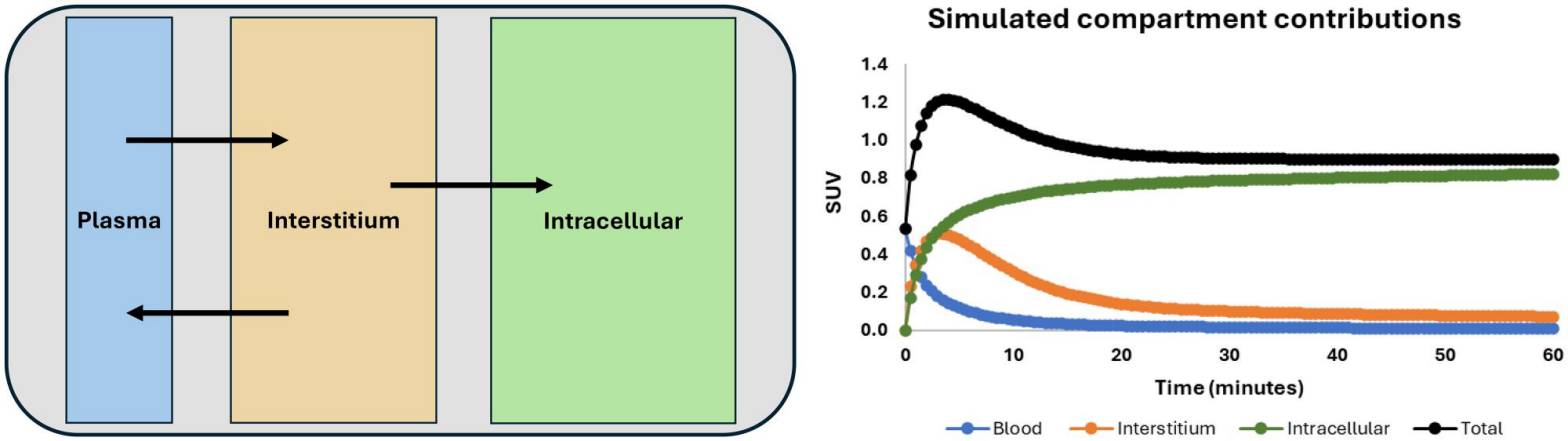
A simple compartment model with distribution to interstitial space and irreversible capture intracellularly. Graph illustrates the temporal course of the contributions.

Another key component of analysis/modelling in the field of nuclear medicine is the use of compartmental analysis (Fig. 9). Compartmental analysis in PET imaging enhances our understanding of drug behaviour by modelling the distribution and kinetics of radiotracers within different biological compartments. This technique allows for precise quantification of how a drug moves through the body, interacts with tissues, and is metabolized. By accurately measuring the uptake and clearance rates of radiotracers, compartmental analysis provides detailed insights into the pharmacokinetics and pharmacodynamics of a drug. This information is crucial for optimizing dosing regimens, improving drug efficacy and safety, and tailoring treatments to individual patients, ultimately leading to more effective and personalized medical interventions.

In radionuclide clinical PET imaging, these advancements enhance the evaluation of new radiopharmaceuticals by:

- Improving Dosimetry: PK/PD modelling helps predict radiation dose distribution, ensuring safety and efficacy of drugs.
- Enhancing Image Analysis: improved radiochemistry workflows providing higher specific activity.
- Advanced pre-clinical models allow for more accurate interpretation of PET images by correlating radiotracer behaviour with physiological processes.
- Optimizing Dosing: Microdosing, and accurate PK/PD models aid in determining optimal dosing strategies for radiopharmaceuticals, maximizing diagnostic and therapeutic benefits.
- Personalizing Treatment: Integrating PK/PD models with PET imaging data supports personalized treatment plans by predicting individual responses to radiopharmaceuticals.

These technological advancements in PK/PD modelling not only improve the development and evaluation of new drug compounds but also enhance the clinical application and effectiveness of radionuclide PET imaging.

### Addressing the lack of translation of tools to application

Despite decades of research, PET has not yet translated into a broadly used tool to study the immune system clinically or for clinical trials. Comparing to other applications can be useful to interpret the reasons for limited adoption. In neuroscience, PET is well established to characterize tissue and explore drug distribution, and tens of experimental and broadly used tracers are available. In drug development, pre-clinical PET pipelines are typically built to parallel drug discovery programs. In Oncology, the established role of FDG-PET for both clinical routine and in clinical trials (for some tumour types) means awareness of PET is high. Building on this, PET has been used to study a range of radiolabelled drug candidates, mechanistic probes including some immune-specific tracers and a growing adoption of tracers such as those targeting somatostatin receptors or PSMA to support theranostic programs.

In applications areas beyond neuroscience and oncology there is an inertia to use PET. Use of PET within clinical trials is considered costly, complex (particularly for bespoke molecular probes) and radiation dose burden in some patient populations is considered prohibitive.

Incorporation of PET within drug development is a chance to catalyse broader use for immunology applications. Pre-clinical application to early drug pipelines will develop confidence in the biological interpretation of the PET signal, particularly if used in tandem with tissue analyses. Pre-clinical evidence will generate interest to invest in clinical methods, creating a translational pipeline like that established in neuroscience. Beyond bespoke PET imaging of labelled assets, a broader molecular probe toolset is required to support drug pipelines. Translational exemplars will demonstrate the value PET can bring to programs and increased organizational experience of deploying PET will reduce reluctance to its use.

Greater use of PET to study the immune system can be achieved through the following:

- Incorporation of methods early in drug development with a focus pre-clinically and translation to early phase trials.
- Demonstrating the value of PET to support drug development decision-making using case studies.
- The use of Total Body PET in order to achieve high image quality with manageable radiation dose burden.
- Clinical exemplars demonstrating the unique insights through whole-body interrogations of systemic diseases.
- A broader range of clinical probes relevant to current immune targeting drug pipelines.

Drug development represents an opportunity to generate a sustained use of PET to study the immune system. In time, some of the methods used may translate into broader diagnostic and treatment monitoring use.

## Conclusions

The toolset available to study the immune system continues to expand, enabling information-rich characterization of tissue and through scalable blood-based biomarkers. Whole-body molecular imaging will deliver an important addition to the toolset. Although spatial resolution is limited and the measurement is defined by a single tracer, PET uniquely delivers a non-invasive, quantitative whole-body evaluation.

Three important elements of the PET experiment will promote adoption: (i) A broadening set of molecular probes translating to clinical use, (ii) High-sensitivity TotalBody scanners will deliver high image quality whilst balancing radiation dose burden, (iii) Decades of analysis and modelling development will provide quantitative parameters from tissues. Each of these elements are combining to provide unique insights into systemic disease, capturing heterogeneity that cannot be measured with other techniques. If tissue, blood, and imaging-based insights are considered in an integrated way, together they will enable delivery of the next generation of diagnostics and personalized therapies to ultimately improve patient outcomes.
